# A suitable and efficient optimization system for the culture of *Chlamydia trachomatis* in adult inclusion conjunctivitis

**DOI:** 10.1093/femspd/ftae020

**Published:** 2024-08-29

**Authors:** Yuan Wei, Xizhan Xu, Leying Wang, Qiankun Chen, Jinsong Li, Xiafei Liu, Zhenyu Wei, Jinding Pang, Yan Peng, Xiaoyan Guo, Zhen Cheng, Zhiqun Wang, Yang Zhang, Kexin Chen, Xinxin Lu, Qingfeng Liang

**Affiliations:** Beijing Institute of Ophthalmology, Beijing Tongren Eye Center, Beijing Tongren Hospital, Capital Medical University, Beijing 100005, China; Beijing Institute of Ophthalmology, Beijing Tongren Eye Center, Beijing Tongren Hospital, Capital Medical University, Beijing 100005, China; Beijing Institute of Ophthalmology, Beijing Tongren Eye Center, Beijing Tongren Hospital, Capital Medical University, Beijing 100005, China; Beijing Institute of Ophthalmology, Beijing Tongren Eye Center, Beijing Tongren Hospital, Capital Medical University, Beijing 100005, China; Diarrhoeal Laboratory, Institute of Viral Diseases, Chinese Center for Disease Control and Prevention, 155 Changbai Road, Changping District, Beijing 102206, China; Diarrhoeal Laboratory, Institute of Viral Diseases, Chinese Center for Disease Control and Prevention, 155 Changbai Road, Changping District, Beijing 102206, China; Beijing Institute of Ophthalmology, Beijing Tongren Eye Center, Beijing Tongren Hospital, Capital Medical University, Beijing 100005, China; Beijing Institute of Ophthalmology, Beijing Tongren Eye Center, Beijing Tongren Hospital, Capital Medical University, Beijing 100005, China; Beijing Institute of Ophthalmology, Beijing Tongren Eye Center, Beijing Tongren Hospital, Capital Medical University, Beijing 100005, China; Beijing Institute of Ophthalmology, Beijing Tongren Eye Center, Beijing Tongren Hospital, Capital Medical University, Beijing 100005, China; Beijing Institute of Ophthalmology, Beijing Tongren Eye Center, Beijing Tongren Hospital, Capital Medical University, Beijing 100005, China; Beijing Institute of Ophthalmology, Beijing Tongren Eye Center, Beijing Tongren Hospital, Capital Medical University, Beijing 100005, China; Beijing Institute of Ophthalmology, Beijing Tongren Eye Center, Beijing Tongren Hospital, Capital Medical University, Beijing 100005, China; Beijing Institute of Ophthalmology, Beijing Tongren Eye Center, Beijing Tongren Hospital, Capital Medical University, Beijing 100005, China; Beijing Institute of Ophthalmology, Beijing Tongren Eye Center, Beijing Tongren Hospital, Capital Medical University, Beijing 100005, China; Beijing Institute of Ophthalmology, Beijing Tongren Eye Center, Beijing Tongren Hospital, Capital Medical University, Beijing 100005, China

**Keywords:** adult inclusion conjunctivitis, *Chlamydia trachomatis*, sexually transmitted disease, ompA genotyping

## Abstract

The prevalence of *Chlamydia trachomatis* infection in the genitourinary tract is increasing, with an annual rise of 9 million cases. Individuals afflicted with these infections are at a heightened risk of developing adult inclusive conjunctivitis (AIC), which is commonly recognized as the ocular manifestation of this sexually transmitted infection. Despite its significant clinical implications, the lack of distinctive symptoms and the overlap with other ocular conditions often lead to underdiagnosis or misdiagnosis of AIC associated with *C. trachomatis* infection. Here, we established six distinct *C. trachomatis* culture cell lines, specifically highlighting the MA104 N*V cell line that exhibited diminished expression of interferon regulatory factor 3 (IRF3) and signal transducer and activator of transcription 1 (STAT1), resulting in reduced interferons. Infected MA104 N*V cells displayed the highest count of intracytoplasmic inclusions detected through immunofluorescence staining, peaking at 48 h postinfection. Subsequently, MA104 N*V cells were employed for clinical screening in adult patients diagnosed with AIC. Among the evaluated cohort of 20 patients, quantitative PCR (qPCR) testing revealed positive results in seven individuals, indicating the presence of *C. trachomatis* infection. Furthermore, the MA104 N*V cell cultures derived from these infected patients demonstrated successful cultivation and replication of the pathogen, confirming its viability and infectivity. Molecular genotyping identified four distinct urogenital serovars, with serovar D being the most prevalent (4/7), followed by E (1/7), F (1/7), and Ia (1/7). This novel cellular model contributes to studies on *C. trachomatis* pathogenesis, molecular mechanisms, and host–pathogen interactions both *in vitro* and *in vivo*. It also aids in acquiring clinically relevant strains critical for advancing diagnostics, treatments, and vaccines against *C. trachomatis*.

## Teaser

Observation of IF staining of MA104 N*V cells 48 h after infection with Chlamydia trachomatis.

## Introduction

Trachoma, an ancient infectious keratoconjunctivitis, was the leading preventable infectious blinding eye disease globally, especially in developing countries (Phillips 2019). In 2002, the World Health Organization reported that there were about 84 million people suffering from active trachoma worldwide (Mariotti et al. 2009, Taylor et al. 2014). This condition is caused by *Chlamydia trachomatis* infection, a significant pathogen also implicated in sexually transmitted diseases (Pekmezovic et al. 2019). It is noteworthy that, despite a notable decline in trachoma incidence due to improved sanitation in recent decades, there has been a surge of 9 million new cases per year in urogenital *C. trachomatis* infections. Individuals with this infection face an elevated risk of adult inclusion conjunctivitis (AIC) (Darville and Hiltke 2010, Mohamed-Noriega et al. 2015). AIC is generally considered to be the ocular manifestation of this sexually transmitted infection (Postema et al. 1996). Clinically, AIC typically presents as chronic follicular conjunctivitis, characterized by an acute or subacute onset that may persist for months if left untreated (Stenberg and Mårdh 1990). The absence of pathognomonic symptoms often results in the underdiagnosis or misinterpretation of AIC associated with *C. trachomatis* infection.

Laboratory tests are essential to determine the causative agent and confirm the clinical diagnosis, including Giemsa staining, direct fluorescent antibody (DFA) test, polymerase chain reaction (PCR), and cell culture (Cook et al. 2005). The nucleic acid test is widely utilized for detecting urogenital *C. trachomatis* infection and chlamydial pneumonia due to its high diagnostic sensitivity (Solomon et al. 2004, Ozüberk et al. 2013). Nevertheless, laboratory diagnosis of AIC is challenging due to the limited specimen, often leading to minimal amounts of nucleic acids within clinical samples. Conventional methods such as PCR, cell culture, and DFA testing have exhibited remarkably low rates of positive results. In a specific study, only 4 (8%) out of 50 conjunctival swab specimens tested positive using DFA, while only 1 (2%) sample yielded positive results through cell culture and PCR techniques (Solomon et al. 2004). Therefore, *in vitro* culture of *C. trachomatis* represents a promising approach to improve the diagnostic sensitivity for AIC, serving as the gold standard for *C. trachomatis* diagnosis and providing the foundation for *in vitro* drug susceptibility test and further investigations of *C. trachomatis* pathogenesis (Di Pietro et al. 2019). Moreover, the development of vaccines relies on live *C. trachomatis* strains. Proper location and correct operation of biopsy, and exploring an optimal *C. trachomatis* culture system are fundamental to *C. trachomatis* culture and achieving accurate diagnosis of AIC.

*Chlamydia trachomatis* is a strictly intracellular parasite that can only grow and replicate within host cells and cannot be cultured *in vitro* (Chiarelli et al. 2020). It can be classified into 20 genotypes based on the outer membrane gene (ompA), which is typically associated with distinct clinical presentations. Genotypes A–C (variant Ba) cause trachoma, while genotypes D–K (variant Da, Ga, and Ia) are linked to urogenital infections, and L1–L3 (variant L2b and L2c) genotypes cause lymphogranuloma venereum. Successful isolation of *C. trachomatis* relies on permissive cell lines, primarily epithelial cells like McCoy, HeLa, African green monkey kidney cells (Vero), and Buffalo green monkey kidney cells (BGMK). MA104 cells, an epithelial cell derived from African green monkey kidney and known for their efficient culture of enteroviruses, have diminished capacity of producing interferon (IFN) upon infection (Lee et al. 2013, Feng et al. 2019, Nurdin et al. 2023). Therefore, MA104 cells may be used to culture *C. trachomatis* because it can elicit an innate immune response similar to that of viruses.

IFN signaling pathway plays a pivotal role in inhibiting the proliferation and spread of *C. trachomatis*, acting indirectly by depleting intracellular tryptophan reserves and stimulating indoleamine-2,3-dioxygenase activity to restrict the tryptophan supply of *C. trachomatis* (Islam et al. 2018, Wang et al. 2022). A notable disparity between urogenital and ocular *C. trachomatis* strains lies in the presence of tryptophan synthetase, enabling urogenital strains to facilitate tryptophan synthesis through indole and sustain their normal life cycle (Caldwell et al. 2003, Carlson et al. 2004). Despite MA104 cells having a blunted IFN response, making them highly permissive for chlamydial replication, we hypothesize that deregulation of IFN signaling in MA104 cells may further enhance chlamydial replication and rescue (Whitaker and Hayward 1985). Coincidentally, a genetically modified MA104 cell line (termed MA104 N*V) was recently established for efficient isolation of rotaviruses, utilizing parainfluenza virus 5 (PIV5, formerly SiV5) V protein and bovine viral diarrhea virus (BVDV) N protease to targeted degradation of signal transducer and activator of transcription 1 (STAT1) and interferon regulatory factor 3 (IRF3), respectively (Rothfuchs et al. 2006, Peterhans and Schweizer 2013). Studies have shown that IRF3 is involved at an early stage in the synthesis of IFN-β induced by *C. trachomatis* (Hu et al. 2015). The JAK/STAT signaling pathway plays a crucial role in defending against viral and intracellular bacterial infections, with human pathogens like the hepatitis C virus targeting STAT1 and STAT2 signaling to evade the immune system (Foy et al. 2003, Rodriguez and Horvath 2004). In addition, upregulation of STAT1 has been demonstrated to inhibit the growth of *C. trachomatis* (Lad et al. 2005). At present, a growing number of AIC caused by *C. trachomatis* genotypes D–K have been reported, but few ocular isolates have been isolated due to limited specimens (Mohamed-Noriega et al. 2015, Petrovay et al. 2015). We propose that the culture efficiency of *C. trachomatis* can be greatly enhanced with the genetically modified MA104 N*V cell line.

In this study, we conducted a comparative analysis of the culturing efficacy of HeLa, HCEC, Vero, BGMK, MA104, and MA104 N*V cells across various bacterial concentrations and time points. The sensitivity of MA104 N*V cells for *C. trachomatis* culture was assessed *in vivo* by quantifying intracytoplasmic inclusions using immunofluorescent (IF) staining, and *in vitro* by quantitative PCR (qPCR). To further elucidate the potential clinical applications of MA104 N*V cell, through a comparative analysis with traditional smear cytology and qPCR, we observed that MA104 N*V cell line demonstrated enhanced proficiency in culture identification, subsequent serotyping, and research applications. Expanding upon these results, we initially conducted serotype analysis on adult patients afflicted with AIC in northern China.

## Methods

### Cell lines

The establishment of the MA104 N*V cell line was facilitated through collaboration with the Institute of Virology, Chinese Center for Disease Control and Prevention, which kindly provided the MA104 and MA104 N*V cell lines. The MA104 N*V cell line was engineered through the incorporation of the V protein derived from para-influenza virus 5 (formerly referred to as SiV5) and the BVDV N protease. This strategic modification enabled the targeted inhibition of the STAT1 and IRF3 cellular signaling pathways (Sánchez-Tacuba et al. 2020). In addition, we prepared HeLa, HCEC (from ATCC), Vero and BGMK cells (from the China Institute of Veterinary Drug Inspection). All cell lines were cultured in Dulbecco’s modified Eagle’s medium supplemented with 10% fetal bovine serum and 1 × Penicillin–Streptomycin solution (Gibco, USA). To stabilize the MA104 N*V cell lines, 1 µg/ml puromycin (Beyotime Biotechnology, China) was added for selective removal of other normal MA104 cells, followed by incubation at 37°C with 5% CO_2_.

### Identification of MA104 N*V cell line

#### IF staining

MA104 and MA104 N*V cells were cultured in 12-well plates at the same concentration for 24 h. Then, IF staining was performed using primary rabbit antihuman IRF3 antibody (#11904S, Cell Signaling Technology, USA; 1:500), rabbit antihuman STAT1 antibody (#14994S, Cell Signaling Technology; 1:500), followed by a secondary goat antirabbit IgG H&L (Alexa Fluor 488; ab150077; Abcam, USA) antibody. Nuclei were counterstained with DAPI (C10005, Beyotime Biotechnology), and representative images were captured using an Olympus microscope (Olympus BX-51, Olympus, Tokyo, Japan).

### RNA extraction and RT-qPCR

Total RNA was extracted from two cell lines, MA104 and MA104 N*V, using the RNeasy Micro Kit (QIAGEN, Hilden, Germany). RNA was reverse transcribed into cDNA using the PrimeScript RT reagent Kit. Quantitative real-time PCR was conducted with LightCycler 480 SYBR GreenⅠ Master Mix (Roche, Basel, Switzerland) on a LightCycler 480 system (Roche). Relative expression was normalized to GAPDH and calculated via the 2^−ΔΔCt^ method. The real-time PCR protocol consisted of an initial denaturation step at 95°C for 3 min, followed by 40 cycles of denaturation at 95°C for 10 s and annealing/extension at 60°C for 30 s. This was succeeded by a final cycle of denaturation at 95°C for 1 min, annealing at 55°C for 30 s, and a final denaturation at 95°C for 30 s. The sequences of the primers used for amplification were as follows: GAPDH forward, 5′-GGAGCGAGATCCCTCCAAAAT-3′ and reverse, 5′-GGCTGTTGTCAACTTCTCATGG-3′; IRF3 forward, 5′-AGAGGCTCGTGATGGTCAAG-3′; and reverse, 5′-AGGTCCACAGTATTCTCCAGG-3′; and STAT1 forward, 5′-CAGCTTGACTCAAAATTCCTGGA-3′; and reverse, 5′-TGAAGATTACGCTTGCTTTTCCT-3′.

### Western blotting

For proteins involved in the IFN expression pathway, the following primary antibodies were used: IRF3 (1:1000), STAT1 (1:1000), and GAPDH (#1E6D9, Proteintech, USA; 1:2000). Protein samples were separated on 10% PAGE gels, transferred to a polyvinylidene fluoride membrane via wet transfer, and blocked with 5% skim milk for 1 h. Subsequently, membranes were incubated overnight at 4°C with primary antibodies. After thrice washes with Tris-buffered saline with Tween-20, the membranes were incubated with goat antirabbit IgG H&L (HRP; ab205718; Abcam; 1:5000) antibody at room temperature for 1 h. The results were detected using enhanced chemiluminescence (Analysis Quiz, Beijing, China).

### *Chlamydia trachomatis* infections

In this study, *C. trachomatis* Type D standard strain (NCBI number CP002054.1) was used to sieve out the optimal method for *C. trachomatis* culture. Cells were seeded onto 12-well plates at a density of 1 × 10 × 5 cells per well and incubated overnight. The resulting confluent monolayers were inoculated with *C. trachomatis* Type D. Two different concentrations of bacterial solutions [1000 inclusion-forming units (IFU) and 5000 IFU] were prepared for cell infection. The plates were incubated 1 h in 37°C and further centrifuged at 350 × *g* for 0.5 h. Then, the inoculum was replaced with fresh medium supplemented with 1 mg/l cycloheximide and the studied compounds or vehicle control.

### IF staining

To observe intracellular inclusion bodies after 24, 48, and 72 h, IF staining was applied. For IF staining, the cells were fixed in 4% paraformaldehyde at room temperature for 10 min. FITC-labeled monoclonal antibody (1:500; ab21211; Abcam) against the major *C. trachomatis* outer membrane protein was added overnight at 4°C. Negative control was incubated without any primary antibody. The cells were followed by staining with DAPI (catalog C10005; Beyotime Biotechnology) and observed under a fluorescence microscope. Representative images were captured with an Olympus microscope (Olympus BX-51, Olympus).

### Quantifying bacterial load

Intracytoplasmic inclusions were counted using IF staining on an inverted microscope using a 40x objective and 20 randomly selected incubation fields after 24, 48, and 72 h of incubation. Mean values were calculated and compared across different culture systems. The infected cell cultures were collected 24, 48, and 72 h after infection, and the genome copy number of *C. trachomatis* was determined by qPCR.

### DNA extraction and qPCR

Following 24, 48, and 72 h of incubation, *C. trachomatis* bacterial fluid was harvested, underwent a freeze–thaw cycle, and was subsequently centrifuged at 500 × *g* for 10 min at 4°C. The resulting pellet was resuspended in 2 ml of sucrose/phosphate/glutamate buffer and aliquoted before storage at −80°C. DNA was extracted using the DNeasy Blood & Tissue Kit (69504; QIAGEN). Quantitative real-time PCR analyses were conducted using the LightCycler 480 SYBR Green I Master Mix on a LightCycler 480 system (Roche). Relative expression levels were normalized to GAPDH using the *2^−ΔΔCt^* method. HeLa cells were utilized as the reference standard for comparing the *2^−ΔΔCt^* ratios among various cell types in statistical analyses. Primer sequences used for amplification included: GAPDH, forward 5′-GGAGCGAGATCCCTCCAAAAT-3′, reverse 5′-GGCTGTTGTCAACTTCTCATGG-3′; and *C. trachomatis*, forward 5′-GCCGCTTTGAGTTCTGCTTCCTC-3′, reverse 5′-ATTTACGTGAGCAGCTCTCTCAT-3′.

### Prospective validation of clinical patients

#### Sample collection and culture

**Figure 1.**
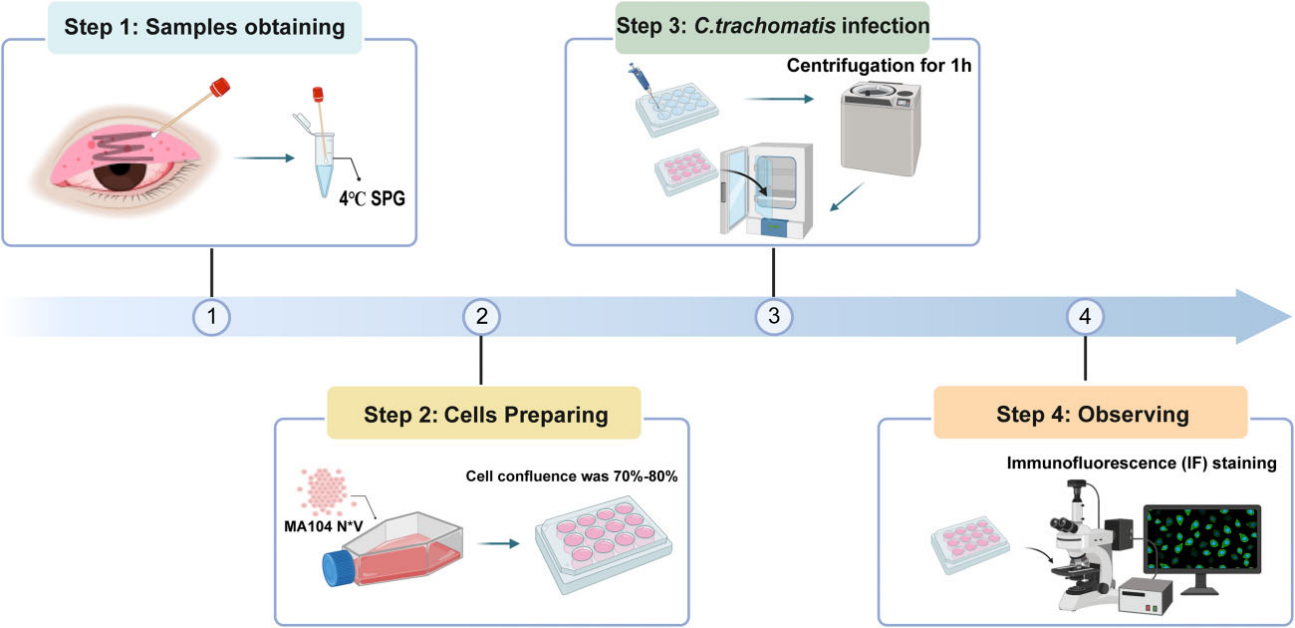
Schematic diagram of conjunctival sac swab sampling, transport, storage, and culture in clinical patients.

From February 2023 to December 2023, 20 patients suspected of AIC were examined at Beijing Tongren Hospital based on routine screening criteria, such as failure of local treatment, chronic symptoms, clinical signs of follicular conjunctivitis, and sexual activity in young individuals. This project was approved by the Medical Ethics Committee (TRECKY2021-024). Conjunctival swabs were collected and sent to the microbiology laboratory of Beijing Institute of Ophthalmology within 24 h for PCR, sequencing, and in vitro expansion using both conventional and optimal culture methods. The standardized processes from sampling to detection and observation was established (Fig. 1). For the culture of *C. trachomatis*, the same six cells were used for the validation and for the comparison. Giemsa staining and IF staining were performed 48 h after inoculation to observe the morphology and structure of the intracytoplasmic inclusions in cell culture. The validation of sensitive cell lines and the *C. trachomatis* culture process has been confirmed based on the positive rate of the clinical samples.

### *Chlamydia trachomatis* genotyping and phylogenetic analysis

The DNA was extracted by DNeasy Blood & Tissue Kit (69504, QIAGEN). The *C. trachomatis* was detected by qPCR to identify the diagnosis. Nested *C. trachomatis ompA* amplification was performed using the Taq DNA enzyme (MT201-02; Biomed Company, China), with primer pairs amplifying a DNA fragment containing the *ompA* gene from all *C. trachomatis ompA* genotypes. 5 µl of DNA was used for the first amplification with primers Yang 1 (GCCGCTTTGAG–TTCTGCTTCCTC) and Yang 2 (ATTTACGTGAGCAGCTCTCTCAT). 3 µl of product from this first round was then amplified using primers Yang 3 (TGACTTTGTTTCGACCGTGTTTT) and Yang 4 (TTTTCTAGATTTCATCTTGTTCAAT/CTG). The amplification conditions for the first round and the nested PCRs were 94°C for 3 min, 40 cycles of 94°C for 30 s, 56°C for 1 min, and 72°C for 1 min and a final extension step at 72°C for 10 min. The final PCR products at 785 bp were analysed on a 1% agarose gel using GoldenView™ nucleic acid gel stain (EL105; Biomed Company). After sequencing, BLAST searches provided by NCBI were used to compare the consensus sequences with known *C. trachomatis* strain sequences. Use MEGA11 software to enter Sequence alignment and phylogenetic analysis were performed. The phylogenetic tree was constructed using the ClustalW model and the maximum-likelihood method in MEGA11. The online tool ITOL was used to edit the phylogenetic tree.

### Statistical analysis

The data were presented as the mean ± standard deviation from at least three independent experiments, and analysed using GraphPad Prism 8.0 software (GraphPad, USA). Normality of quantitative data was assessed using the Shapiro–Wilk test. Differences in continuous variables between the two groups were assessed using Student’s *t*-test or Mann–Whitney test, respectively. Between the three groups, one-way ANOVA or Kruskal–Wallis test was used. Correlations between the number of intracytoplasmic inclusions and the *C. trachomatis* DNA copy number were examined using Pearson’s correlation analysis. Statistical significance was defined as *P* < .05 when comparing the two indicated settings.

## Results

### Characterization of six cell lines for *C. trachomatis* infection

**Figure 2.**
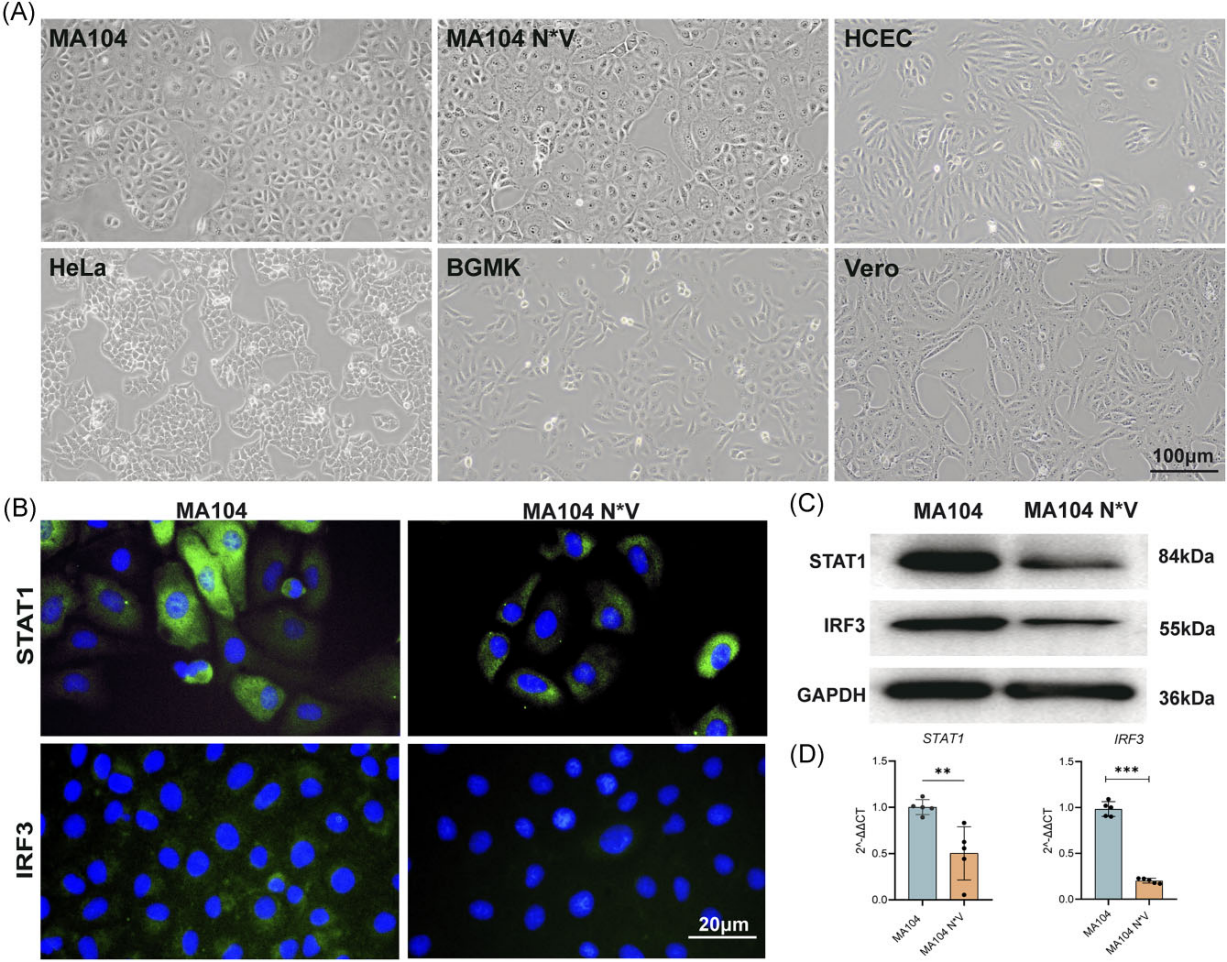
Growth and characterization of six epithelial cell lines for culture of *C. trachomatis*. (A) Morphology of MA104, MA104 N*V, HCEC, Hela, BGMK, and Vero cells under contrast phase microscope. Scale bar = 100 µm. (B) Immunofluorence staining of STAT1 and IRF3 within MA104 and MA104 N*V cells. Nuclei were counterstained with DAPI. Scale bar = 20 µm. (C) Western blotting demonstrating the decreased expression of STAT1 and IRF3 in MA104 N*V cells. GAPDH was used as a loading control. (D) RT-qPCR showing the significantly decreased expression levels of STAT1 and IRF3 in the MA104 N*V cell line (**P < .01, ***P < .001).

To systematically screen suitable cell line for *C. trachomatis* infection, six epithelial cell lines, including MA104, MA104 N*V, HCEC, HeLa, BGMK, and Vero cells, were seeded directly into the 75 cm^2^ culture flask. All cell lines showed homogenous epithelial cell morphology and adherent growth without detectable contamination, as evidenced by morphological features (Fig. 2A). IF staining demonstrated a significant reduction in the expression levels of IRF3, STAT1 in MA104 N*V cells compared to MA104 cells (Fig. 2B). These findings were further validated by western blotting analysis, indicating a significant disparity in IRF3 and STAT1 protein levels between the wild-type MA104 and MA104 N*V cells (Fig. 2C). RT-qPCR results also confirmed a significant downregulation of both IRF3 and STAT1 in MA104 N*V cells compared to the wild-type strain (both *P* < .05; Fig. 2D). Taken together, these observations collectively suggest that MA104 N*V cells exhibit reduced endogenous levels of STAT1 and IRF3, making them the promising cell line for isolating and culturing clinical *C. trachomatis* strains.

### Evaluation of the efficiency of culturing *C. trachomatis* in different cell line*s*

**Figure 3.**
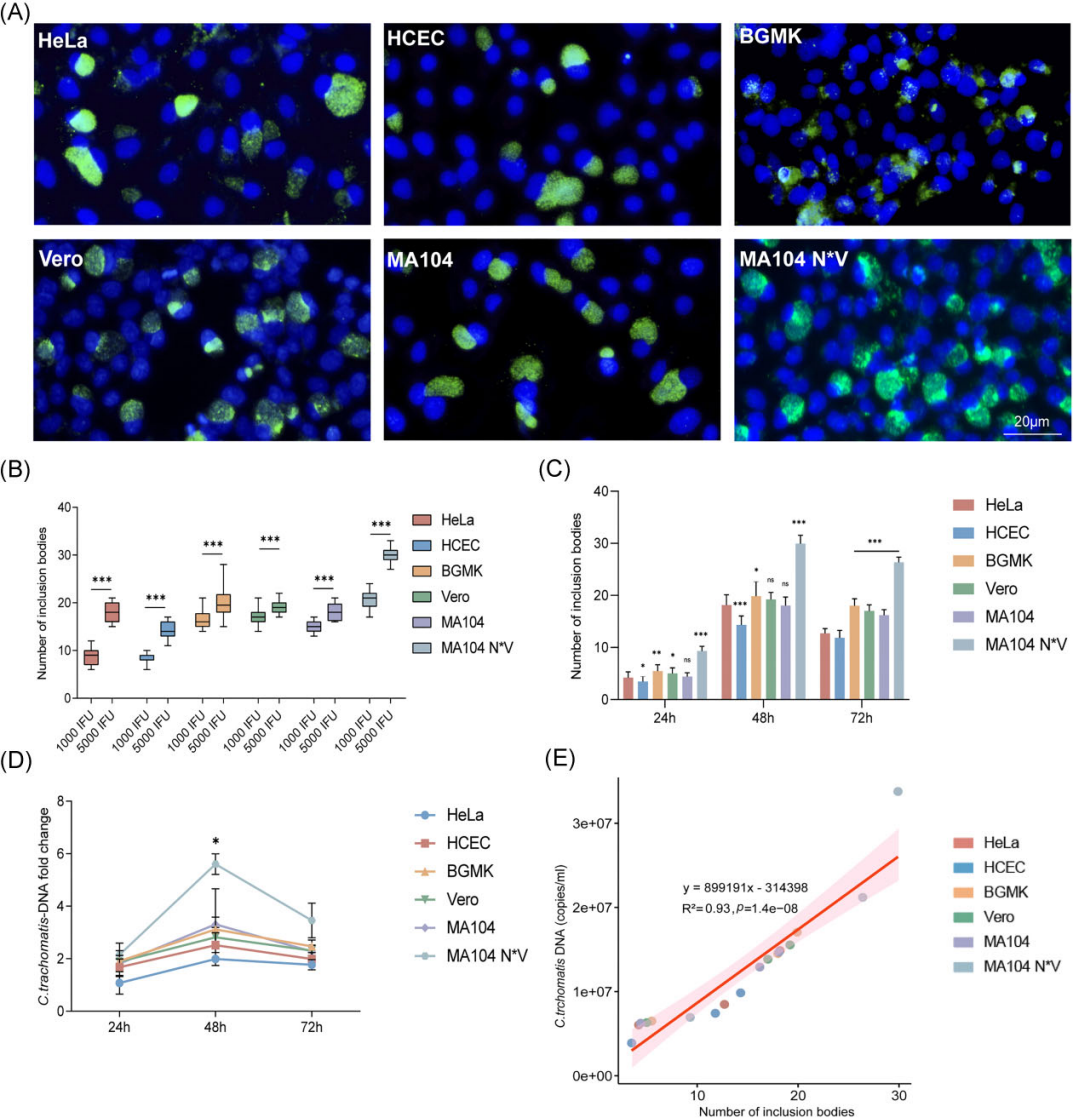
Assessments of infectivity of *C. trachomatis* across six epithelial cell lines. (A) Observation of six distinct cell lines 48 h post *C. trachomatis* infection using IF staining. Scale bar = 20 µm. (B) Comparison of the number of intracytoplasmic inclusions in six different cell lines infected with 1000 and 5000 IFU of *C. trachomatis*. The number of intracytoplasmic inclusions were assessed in 20 randomly selected fields at 48 h postinfection (***P < .001). (C) Number of intracytoplasmic inclusions in cells infected with 5000 IFU of *C. trachomatis*. At 24, 48, and 72 h postinfection, 20 randomly selected fields were assessed for the number of intracytoplasmic inclusions in IF staining (comparison with HeLa cells, *P < .05, **P < .01, ***P < .001, and ns: nonsignificant). (D) *C. trachomatis* cultures were harvested and quantified using qPCR at 24, 48, and 72 h. Statistical comparison with HeLa cells revealed significant differences (*P < .05). (E) Scatterplot showing the strong positive correlation between the number of intracytoplasmic inclusions and the *C. trachomatis* DNA copy number. Pearson correlation was used.

Next, the *C. trachomatis* standard strain (genotype D) was used to infect six cell lines *in vitro* to evaluate the infection efficiency of different culture systems. In all six cell lines (Fig. 3A), as visualized by IF staining, *C. trachomatis* proliferated and formed typical intracellular inclusions, with notably extensive formation in MA104 N*V cells. To determine the optimal inoculating dose, cells were infected with 1000 IFU and 5000 IFU of *C. trachomatis*, respectively. Notably, at an initial infection concentration of 5000 IFU, the number of inclusions in all six cell types was significantly higher than that in the 1000 IFU wells (*P* < .001, Fig. 3B). To reveal the temporal dynamics of intracytoplasmic inclusions during *C. trachomatis* infection, infected cells were harvested at 24, 48, and 72 h postinfection, and the number of intracytoplasmic inclusions was quantified under light microscopy. Results showed that the number of intracytoplasmic inclusions in all cell lines exhibited a trend of first increasing and then decreasing, reaching a peak at 48 h. At an initial inoculating dose of 5000 IFU, the number of intracytoplasmic inclusions in the MA104 N*V cell line peaked at 48 h, with an average of 29.9 ± 1.6 inclusions per field of view, significantly higher than the number in HeLa cells (18.2 ± 2.0 inclusions/field), HCEC cells (14.3 ± 2.0 inclusions/field), BGMK cells (19.9 ± 2.9 inclusions/field), Vero cells (19.2 ± 1.4 inclusions/field), and MA104 cells (18.1 ± 1.6 inclusions/field; Fig. 3C). Detailed data on the dynamic changes in the number of intracytoplasmic inclusions are shown in Table 1. Employing qPCR for more precise quantification, the relative amount of *C. trachomatis* was normalized to cellular GAPDH levels. Notably, the MA104 N*V cell line consistently exhibited the highest *C. trachomatis* DNA copy number across all three assessed time points (Fig. 3D). Additionally, Pearson correlation analysis was performed between the number of intracytoplasmic inclusions and the *C. trachomatis* DNA copy number. There’s a strong positive correlation between the two indicators (linear regression: y = 899 191*x + 314 398; *R*^2^ = 0.93, *P* < .01), indicating that the number of intracytoplasmic inclusions can be used as a surrogate indicator for *C. trachomatis* DNA copy number (Fig. 3E). Therefore, the number of intracytoplasmic inclusions will be used as a quantification of pathogens hereafter. Collectively, these data indicate that MA104 N*V cells are the most sensitive cell line and have the highest infection efficiency.

**Table 1.** Comparison of inclusion body counts of six kinds of cells infected with *C. trachomatis* at 24, 48, and 72 h under IF staining.

	1000 IFU			5000 IFU		
Cell lines	24 h	48 h	72 h	24 h	48 h	72 h
HeLa	3.0 ± 0.8	9.2 ± 2.1^b^	8.6 ± 1.3	4.2 ± 1.2^a^	18.2 ± 2.0^a,b^	12.7 ± 1.4
HCEC	2.5 ± 0.7	8.9 ± 1.6^b^	6.7 ± 1.0	3.5 ± 1.1	14.3 ± 2.0^a,b^	11.8 ± 1.2^a^
BGMK	3.7 ± 0.9	16.6 ± 1.6^b^	15.8 ± 1.0	5.5 ± 1.2^a^	19.9 ± 2.9^a,b^	18.0 ± 1.3^a^
Vero	3.8 ± 0.8	16.8 ± 1.9^b^	14.9 ± 1.4	5.0 ± 1.1^a^	19.2 ± 1.4^a,b^	17.0 ± 1.2^a^
MA104	3.5 ± 0.8	15.5 ± 1.6^b^	13.8 ± 1.0	4.4 ± 0.8	18.1 ± 1.6^a,b^	16.2 ± 1.6^a^
MA104 N*V	5.3 ± 0.9	20.5 ± 1.5^b^	19.9 ± 1.9	9.3 ± 0.9^a^	29.9 ± 1.6^a,b^	26.4 ± 1.0^a^

a Statistically significant difference in the number of intracytoplasmic inclusions after *C. trachomatis* infection with 1000 and 5000 IFU at the same time point (*P* < .05).

b Statistically significant differences in the number of intracytoplasmic inclusions at 48 h compared to 24 and 72 h postinfection under consistent conditions of initial *C. trachomatis* concentration within the identical cells (*P* < .05).

### Real-world clinical validation of the detection of *C. trachomatis* using MA104 N*V cell line

To further verify the clinical diagnostic value of the modified MA104 N*V cell line, which is highly susceptible to *C. trachomatis*, these cell lines were used to culture the superior and inferior palpebral conjunctival sac specimens obtained from patients with suspected AIC.

**Figure 4.**
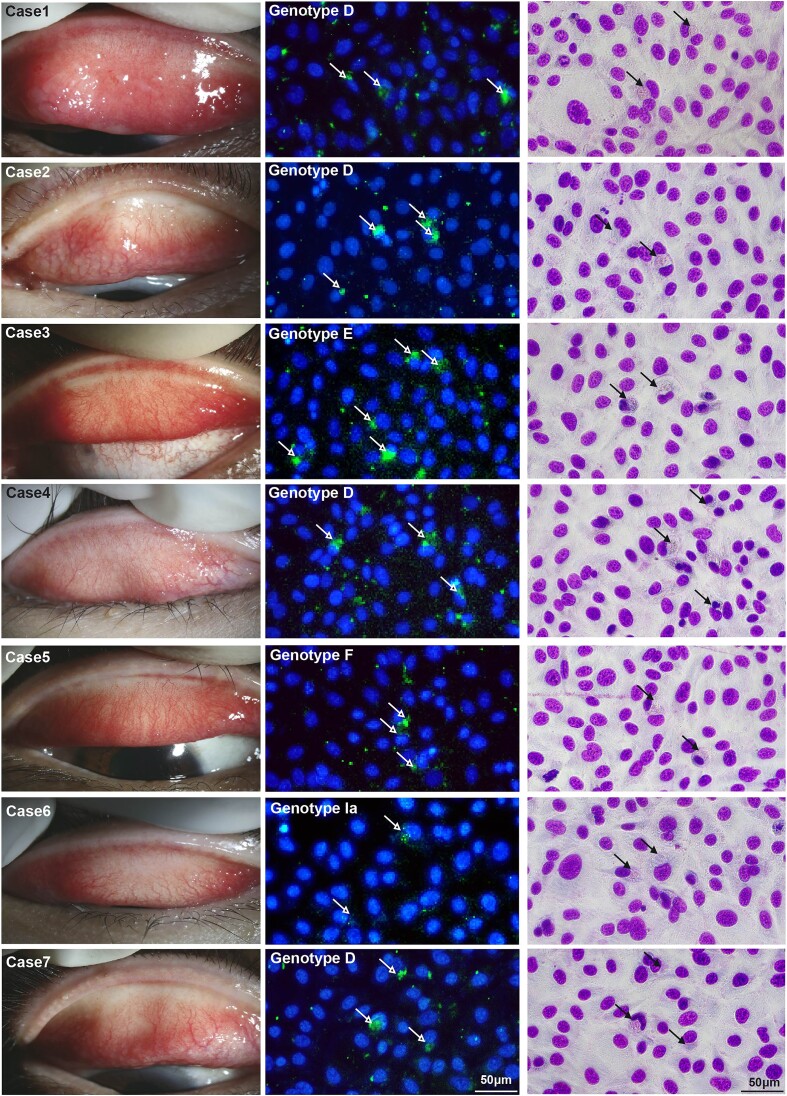
Slit-lamp images from seven confirmed AIC cases, accompanied by laboratory test in MA104 N*V cells with IF and Giemsa staining. The arrow indicates typical *C. trachomatis* intracytoplasmic inclusions detected under the microscope. Scale bar = 50 µm.

A total of 20 patients were included in this study. Demographic, clinical characteristics, and laboratory test results are detailed in Table 2. Briefly, the median age of patients was 35 years (range 23–55 years), 60% (*n* = 12) were male, and the average disease duration was 7.5 weeks. Patients showed edema and thickening of the palpebral conjunctiva, massive hemorrhage, and numerous follicles (80%, *n* = 16), particularly at the medial and lateral canthus. The positive rate of *C. trachomatis* qPCR test was 35.0% (7/20). All seven clinical samples with positive PCR results exhibited detectable intracytoplasmic inclusions in the MA104 N*V cell culture system (Fig. 4). In contrast, only six samples were detected in Hela and BGMK cells, and only five samples were detected in MA104 cells and Vero cells. Following the HCEC cell culture, only four samples were successfully isolated and cultured. In addition, we also evaluated the ratio of neutrophils and lymphocytes in the conjunctival cell scrapings, and found that there were a relatively large number of neutrophils [Median (38.3%)] and a small number of lymphocytes [Median (21.5%)] infiltration. Giemsa and IF staining were performed 48 h after inoculation, and the typical cap-like structure of intracytoplasmic inclusions was found (Fig. 4). Notably, all conjunctival swabs that tested positive for *C. trachomatis* by qPCR also produced positive results in MA104 N*V cells, showing 100% concordance. These results demonstrate that MA104 N*V has demonstrated high practicability in real-world clinical diagnosis and can provide additional laboratory evidence for the clinical diagnosis of AIC.

**Table 2.** Summary of laboratory examination results for 20 patients with suspected AIC.

				Scrape cytology			qPCR	Cell culture						
Patient ID	Gender/age	Duration (weeks)	Follicles/papilla	Neutrophil (%)	Lymphocyte (%)	Iintracytoplasmic inclusion	*C. trachomatis*	HeLa	HCEC	BGMK	Vero	MA104	MA104 N*V	Genotype
Case 1	M/35	8	+	45	25	+	+	−	−	+	+	+	+	D
Case 2	F/40	4	+	40	30	+	+	+	+	−	−	+	+	D
Case 3	M/36	4	+	30	25	−	+	+	−	+	+	+	+	E
Case 4	F/42	8	+	35	30	−	+	+	+	+	+	−	+	D
Case 5	M/31	5	+	50	20	−	+	+	+	+	+	+	+	F
Case 6	F/23	6	+	45	15	+	+	+	+	+	+	+	+	Ia
Case 7	M/51	8	−	30	25	+	+	+	−	+	−	−	+	D
Case 8	M/36	3	+	35	20	−	−	−	−	−	−	−	−	−
Case 9	M/25	8	+	45	15	−	−	−	−	−	−	−	−	−
Case 10	M/49	11	−	40	20	−	−	−	−	−	−	−	−	−
Case 11	M/35	4	+	30	25	−	−	−	−	−	−	−	−	−
Case 12	M/29	12	+	40	10	−	−	−	−	−	−	−	−	−
Case 13	F/55	10	+	40	35	−	−	−	−	−	−	−	−	−
Case 14	M/36	9	+	30	25	−	−	−	−	−	−	−	−	−
Case 15	F/26	7	+	35	20	−	−	−	−	−	−	−	−	−
Case 16	F/34	8	−	40	25	−	−	−	−	−	−	−	−	−
Case 17	M/28	11	+	50	15	−	−	−	−	−	−	−	−	−
Case 18	M/29	6	+	35	20	−	−	−	−	−	−	−	−	−
Case 19	F/34	9	+	40	10	−	−	−	−	−	−	−	−	−
Case 20	F/31	10	−	30	20	−	−	−	−	−	−	−	−	−

### Phylogenetic analyses of isolated *C. trachomatis* strains

**Figure 5.**
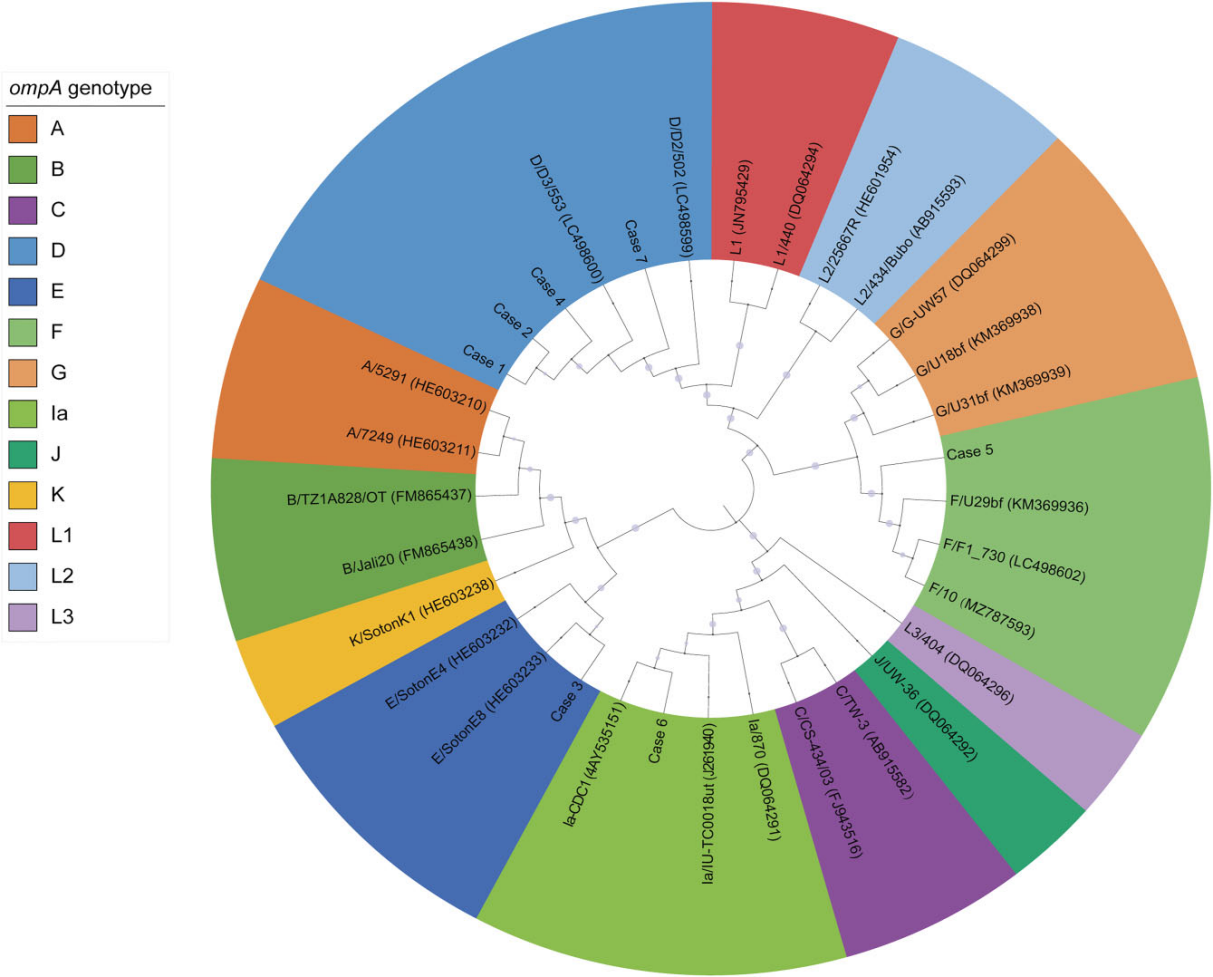
Maximum-likelihood phylogenetic tree displaying the evolutionary relationships of *C. trachomatis ompA* gene sequences. The leaves of the tree are color-coded according to genotype. GenBank accession numbers of all available reference sequences, indicated in parentheses, uniquely identify each sequence in the analysis.

Finally, a total of seven *C. trachomatis* positive samples from patients with AIC were selected to determine their genotypes. All samples were successfully genotyped by PCR amplification, Sanger sequencing, and phylogenetic analysis of *C. trachomatis ompA* gene. The genotype of all samples was first determined by BLAST and then confirmed by the phylogenetic tree constructed by after local alignment analysis (Fig. 5). The predominant genotype was identified as D, comprising 57.1% of the samples, *n* = 4), followed by E, F, and Ia, each representing 14.3% of the strains (*n* = 1 for each). These findings contribute valuable insights into the prevalence and genetic diversity of *C. trachomatis* strains in patients with AIC, with implications for understanding the epidemiological landscape of the infection.

## Discussion

Globally, *C. trachomatis* stands as the most prevalent bacterial sexually transmitted infection, with nearly 131 million new cases diagnosed annually (Kasi et al. 2004, Fenwick 2012). Despite its staggering impact, an estimated 4.2% of females worldwide harbor this infection, with ~80% being asymptomatic, rendering it undiagnosed. The consequences of this undetected infection can lead to various conjunctivitis syndromes, including trachoma, adult and neonatal inclusion conjunctivitis, and lymphogranuloma venereum (Lockington et al. 2013). AIC, caused by *C. trachomatis* genotypes D–K, represents a prevalent ocular manifestation. However, the lack of typical clinical symptoms and resemblance to other ocular diseases, such as allergic conjunctivitis, often results in underdiagnosis or misdiagnosis of AIC associated with *C. trachomatis* infection (Mohamed-Noriega et al. 2015). In this study, we successfully utilized a highly susceptible cell line with reduced IFN expression, MA104 N*V, to culture *C. trachomatis* and diagnose AIC, resulting in enhanced infection efficacy compared to commonly used cell lines. We also optimized infection conditions, including timing, concentration, and staining methods. The utilization of MA104 N*V cells represents a novel approach to study *C. trachomatis* conjunctival infections, holding promise for genome-wide genetic variation analysis and even the development of pathogen-specific vaccines and drugs in the future.

*Chlamydia trachomatis*, as a unique intracellular pathogen, has traditionally been cultured using McCoy, HeLa, BGMK, and Vero cell lines (Talley et al. 1994). However, the cell culture process is time-consuming, labor-intensive, and requires specialized facilities and expertise, posing challenges for broader implementation (Di Pietro et al. 2019). While PCR remains the primary clinical detection method for *C. trachomatis*, only cell culture allows for crucial aspects like drug sensitivity test, genetic studies, and potential vaccine development (Aiyar et al. 2014). The primary goal of our study was to identify an improved cell line for isolation and culture of *C. trachomatis* from limited clinical samples to increase detection rates. In this study, our initial attempts with MA104 and MA104 N*V cells demonstrated that MA104 N*V cells, known for suppressing IFN expression, yielded the highest number of intracytoplasmic inclusions at both low and high inoculum concentrations. Notably, the genetically modified MA104 N*V cells have been widely used in rotavirus culture due to its ability to suppress IFN, a critical component of the host’s defense against various intracellular pathogens (Peterhans and Schweizer 2013, Sánchez-Tacuba et al. 2020). Our findings corroborate that the degradation of STAT1 and IRF3, inhibiting intracellular IFN expression, enhances the infection efficacy of *C. trachomatis* (Rothfuchs et al. 2006). Recently, Carlson et al. also revealed that IFN played an important role in preventing the invasion and proliferation of *C. trachomatis* (Carlson et al. 2004). Meanwhile, it has been reported that in the presence of low concentrations of indole, *C. trachomatis* can evade IFN-γ-mediated clearance, maintaining its pathogenicity (Caldwell et al. 2003, Carlson et al. 2004). This study introduces an innovative approach for diagnosing AIC, successfully isolating and cultivating *C. trachomatis* from clinical samples using the modified MA104 cell line.

Our study also further determined the optimal conditions for culturing *C. trachomatis in vitro*. We found that the peak number of intracytoplasmic inclusions occurred at 48 h postinfection, and MA104 N*V cells demonstrated the highest sensitivity to *C. trachomatis* among six cell lines tested. While Giemsa staining is effective for detecting intracytoplasmic inclusions, immunofluorescence staining proved more precise, especially for limited conjunctival specimens with minimal atypical intracytoplasmic inclusions (Lipkin et al. 1986). Our results emphasized the higher sensitivity of immunofluorescence staining through increased antigen–antibody interaction, although it comes with a higher cost and the need for additional equipment.

The successful validation of MA104 N*V cell line susceptibility using a standard D strain led us to employ this cell line for culturing *C. trachomatis* from clinical conjunctival sac specimens. A standardized workflow was established covering the entire process from sample collection to detection and observation. We also found that MA104 N*V cells are suitable for clinical detection of *C. trachomatis* and is consistent with the results of PCR. Moreover, we have identified four distinct genotypes of *C. trachomatis*, including genotype D, E, F, and Ia, consistent with the epidemiological characteristics of *C. trachomatis* genotypes reported globally (Stenberg and Mårdh 1990, Rao et al. 1996, Malhotra et al. 2013, Mohamed-Noriega et al. 2015). Additionally, our study is the first to document the genotypes with high genetic diversity of adult patients with inclusion conjunctivitis in northern China, which may prompt increased domestic surveillance efforts for *C. trachomatis* infection and provide valuable insights for vaccine development.

Despite these contributions, our study has some limitations. First, we focus on a limited set of *C. trachomatis* isolates (genotype D, E, F, and Ia) due to the availability of clinical samples. These findings may vary with other *C. trachomatis* isolates, and further research is needed to confirm the superiority of the modified MA104 N*V cells. Second, the limited number of patients with conjunctivitis in our ophthalmology-focused hospital restricted the sample size and source for this study.

In conclusion, MA104 N*V cells demonstrated high sensitivity to *C. trachomatis* and proved effective for inoculating clinical conjunctival samples. Immunofluorescence staining emerged as a valuable tool for detecting intracytoplasmic inclusions, with MA104 N*V cells exhibiting the highest intracytoplasmic inclusions count at 48 h postinfection with *C. trachomatis*. Notably, *C. trachomatis* genotype D was predominant in the obtained ocular conjunctival specimens. Our study provides a powerful tool for clinical isolation and culture of *C. trachomatis* and lays a foundation for future research on the genetic diversity of this pathogen based on whole-genome sequencing and vaccine development.
